# Excitement for our future

**DOI:** 10.18632/oncotarget.28116

**Published:** 2021-11-09

**Authors:** John L. Marshall

**Keywords:** multi-omics, molecular signatures, BRCA

***News on:** Opposing effects of BRCA1 mRNA expression on patient survival in breast and colorectal cancer and variations among African American, Asian, and younger patients by Leaf et al. Oncotarget. 2021; 12:1992–2005. https://doi.org/10.18632/oncotarget.28082. [PubMed]*

The manuscript published by El-Deiry et al. is outstanding science as a stand-alone work, providing us results that will undoubtedly prove valuable as we continue our quest to uncover the many secrets of cancer. These data will provoke further research and inquiry into the BRCA molecular mechanisms and relationships underpinning the observations provided by these valuable data sets. Explaining the differences observed between breast cancer and colon cancer will likely be of critical interest to both the basic and clinical science communities.

But as I read this manuscript, I see a much brighter future ahead for all in the cancer research business. First, we must recognize the value and fundamental lessons that our molecular and clinical databases hold within them. The investment we and others have made in amassing high quality, clinically annotated, multi-omic databases is beginning to pay off. Those of us who are clinicians should remember that every time we are grudgingly documenting in our electronic medical records, we are actually contributing critical information that will soon be used in an analysis just like this paper. It is clear we are moving from our infancy of molecular medicine to our adolescence. Where just ten years ago we were ordering single genetic tests on patients, it is now quite routine to do broad multi-omic molecular profiling on the majority of our patients with metastatic disease. These data sets are very quickly providing us with molecular signatures that can make us better and more efficient clinicians, even when it is something as simple as choosing between 2 previously equivalent chemotherapy regimens [1]. It is through these large data sets that we will mature and our practice of medicine will improve with less waste and a better understanding of the right patient with the right drug.

Second, this paper brings forward the point that lessons learned in one cancer type are not always translatable to other cancer types. As we celebrate the annual cancer festival of breast cancer awareness month, we must remember that it is equally important to invest in the research of other cancers. This paper is a clear demonstration of how one molecular observation in breast cancer is the complete opposite in colorectal cancer. Of course, this is disappointing to us as it means the work ahead is that much harder. But it is work that we must continue to pursue on behalf of our patients.

I feel our future is very bright. The decade ahead will likely be our most successful yet as we continue to seek cures, novel therapies, and better biomarker predictors of our patients’ future. We must continue to build shared molecular databases annotated with clinical outcomes. These databases must be of the highest quality and reliability if we are indeed to move from our adolescence to a state of maturity.
